# A Universal Approach Toward Intrinsically Flexible All-Inorganic Perovskite-Gel Composites with Full-Color Luminescence

**DOI:** 10.34133/research.0412

**Published:** 2024-07-08

**Authors:** Dourong Wang, Jingjing Cui, Yang Feng, Yunlong Guo, Jie Zhang, Yaqi Bao, Haoran Deng, Ruiqian Chen, Xinxin Kang, Biao Zhang, Lin Song, Wei Huang

**Affiliations:** ^1^ Frontiers Science Center for Flexible Electronics (FSCFE), Institute of Flexible Electronics (IFE), Ningbo Institute of Northwestern Polytechnical University, Northwestern Polytechnical University, Xi’an 710072, China.; ^2^ Key Laboratory of Flexible Electronics (KLOFE) & Institute of Advanced Materials (IAM), Nanjing Tech University (NanjingTech), Nanjing 211816, China.; ^3^ Key Laboratory for Organic Electronics & Information Displays (KLOEID) and Institute of Advanced Materials (IAM), Nanjing University of Posts & Telecommunications, Nanjing 210023, China.

## Abstract

The combination of all-inorganic perovskites (PVSKs) and polymers allows for free-standing flexible optoelectronic devices. However, solubility difference of the PVSK precursors and concerns over the compatibility between polymer carriers and PVSKs imply a great challenge to incorporate different kinds of PVSKs into polymer matrices by the same manufacturing process. In this work, PVSK precursors are introduced into poly(2-hydroxyethyl acrylate) (PHEA) hydrogels in sequence, in which the PVSK-gel composites are achieved with full-color emissions by simply varying the precursor species. Moreover, it is found that CsBr has a higher interaction energy with the (111) plane of CsPbBr_3_ than the (110) plane; thus, the CsPbBr_3_ crystals with a shape of truncated cube and tetragon are observed during the CsPbBr_3_–Cs_4_PbBr_6_ phase transition over time. The PVSK-gel composites feature excellent bendability, elasticity, and stretchable deformation (tensile strain > 500%), which allows for 3D printing emissive customized stereoscopic architectures with shape-memory features.

## Introduction

All-inorganic perovskites (PVSKs) as a revolutionary optoelectronic material have gained an increasing research interest owing to their tunable bandgap, high photoluminescence quantum yield (PLQY), low exciton binding energy, and excellent thermal endurance [1–6]. These merits promote all-inorganic PVSKs to hold great promise in applications of light-emitting diodes (LEDs) [3,7], photovoltaics [8–11], photoelectric detection [12,13], and imaging displays [14,15]. In the context of these optoelectronic applications, a variety of PVSK-based devices with flexible forms are springing up recently, following the growing demand for the ingenuity and customization in device design, system integration, and smart control [16,17]. To date, the main type of the flexible devices is in the form of nanostructured PVSKs attaching onto the naturally flexible substrates, in which bendability is the main manifestation [18–21]. Therefore, the device flexibility strongly relies on the mechanical properties of the applied substrates. With more demand of high stretching strain, small bending radius in form, and large elastic deformation, developing intrinsic flexible devices is the future technology heading [22]. Along this line, fabricating free-standing composites by incorporating PVSK grains into polymer matrices is a possible choice, which completely exclude the use of flexible substrate [23]. This exclusion allows for a wide variety of manufacturing techniques for flexible devices not limiting to conventional spin coating or slot-die coating, leading to a distinct possibility of designing novel device architectures to optimize the device performance and to meet diversified and personalized requirement [24–26].

To date, extensive research is dedicated to the PVSK-polymer composites with respect to the superior flexibility and designability. Physical blending pre-made PVSK nanocrystals with polymer carriers and mixing PVSK precursor solutions with polymers have been frequently reported in literature for the composite preparation [27,28]. For example, Zhu et al. [29] reported a highly blue-green emissive PVSK powders with the supramolecular assembly octahedral clusters. However, the powders typically need to be synthesized in advance, implying a complex manufacturing process for the composites. The high surface energy tends to induce the agglomeration of PVSK nanocrystals in polymer carriers and thereby a decreased PLQY [30]. As an alternative, Chen and colleagues [31] used the so-called one-pot polymerization method to prepare PVSK-polymer composites, in which PVSK precursor solution [CsBr and PbBr_2_ in dimethylformamide (DMF)] are incorporated into polymethylmethacrylate (PMMA). However, this approach requires a similar solubility for all components of the PVSK-polymer composites, which is a non-negligible limiting factor to explore the product variety. For instance, cesium halides have a poor solubility in ordinary aprotic solvents such as DMF or dimethyl sulfoxide (DMSO) but are easily dissolved in water, while lead halides are exactly the opposite [32]. The solubility difference limits the selectivity of polymer carriers and even the quality of the final product [12,33]. In general, the current approaches for PVSK/polymer composite preparation remain challenging mainly in 2 aspects. First, concerns over compatibility between the PVSK nanocrystals and polymer carriers limit their pairing choice. Second, the marked difference in solubility implies a great challenge in optimizing luminescent performance and flexibility in terms of PVSK loading and crystallization regulation.

Here, we report a novel 2-step method to incorporate PVSK grains into poly(2-hydroxyethyl acrylate) (PHEA) gels to finalize the PVSK-gel composites with independent intrinsic flexibility and excellent luminescent performance. In this approach, relying on a good solubility in water, cesium halides are homogeneously distributed in the PHEA gel, followed by a penetration of lead halide solution to induce in situ crystallization of PVSKs inside the gel matrix. The separate incorporation of PVSK precursors solves the problem regarding the different solubility of cesium halides and lead halides in the same solvent, in which any ratios of these 2 halide salts can be achieved consequently. Through independent regulation of PVSK precursors, the superior luminescent performance and storage stability are achieved, and the phase transition of PVSKs is schematically studied and unraveled. Moreover, for the conventional manufacturing methods such as combining pre-synthesized PVSK crystals with polymers or mixing PVSK precursor solutions with gel monomers, it is necessary to take the consideration of the compatibility between PVSK grains or solution with polymer carriers, which limits the variety of the resultant PVSK-gel composites. In the present work, the incorporation of cesium halides into gels in the first step is independent on the second step of lead halide solution penetration, which allows for the preparation of different PVSK species regardless of the used polymer carriers. As a result, different emission colors can be achieved in the PVSK-gel samples by only changing the halide elements in the PVSK precursors. Combined with 3-dimensional (3D) printing technologies, customized shapes with full-color emissions are demonstrated to give a proof of practicability as the prepared PVSK-gel composites exhibit excellent bendability, elasticity, stretchable deformation (tensile strain > 500%), and shape-memory features.

## Results

### Fabrication of luminescent PVSK-gel composites

**Fig. 1.**
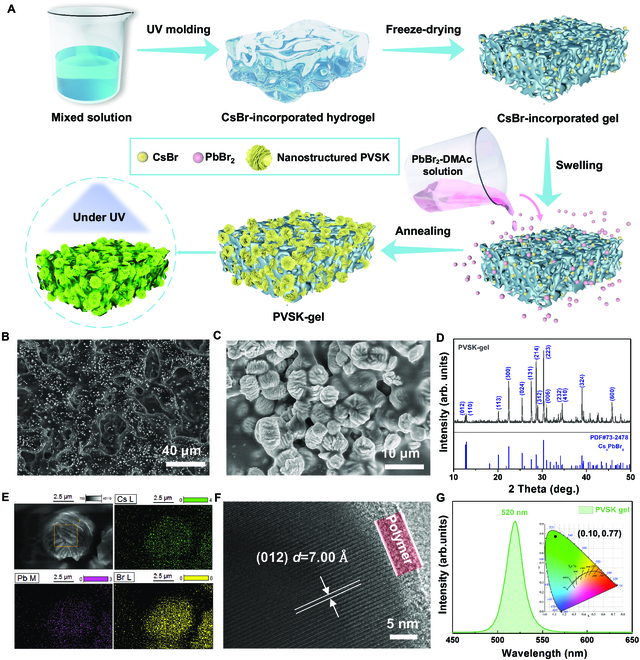
Luminescent PVSK-gel composites. (A) Schematic illustration of the 2-step method to fabricate the luminescent PVSK-gel composite. (B) SEM image of the CsBr-incorporated gel after the water removal. (C) SEM image and (D) XRD curve of the PVSK-gel composite. The vertical dark blue sticks in (D) represent the peak positions and their respective relative intensities of Cs_4_PbBr_6_, taken from The International Centre for Diffraction Data (ICDD). (E) SEM and the corresponding EDS mapping images of the flower-like structure in the PVSK-gel sample. (F) HRTEM image of Cs_4_PbBr_6_ inside the PVSK-gel sample. (G) PL spectrum (λ_Excitation_ = 365 nm) of the PVSK-gel (inset: the color coordinate in the CIE 1931 chromaticity diagram of the PL spectrum).

In this work, the incorporation of PVSK grains into the hydrogel matrix is accomplished through a 2-step method. The complete workflow description is schematically illustrated in Fig. 1A. First, CsBr aqueous solution and HEA monomers are mixed in a certain proportion, which is then cured into a CsBr-incorporated gel with a custom-made shape through an ultraviolet (UV) photopolymerization process. After freeze-drying treatments, the removal of water induces pore formation inside the CsBr-incorporated gel. Afterward, the sample is immersed into a PbBr_2_ solution. The formed pores provide channels for the PbBr_2_ solution penetration, which is essential for the reaction between CsBr and PbBr_2_. Finally, the swollen gel is annealed to assist the PVSK crystallization and to remove the solvents. Up to this point, the flexible luminescent PVSK-gel composite is obtained, in which the chemical structure of the cross-linked gel is schematically shown in Fig. S1.

Figure 1B displays a plan view scanning electron microscopy (SEM) image of the gel with CsBr incorporation after the water removal. The CsBr grains are homogeneously distributed in the gel matrix, which is the consequence of good compatibility between the CsBr aqueous solution and HEA monomers. In addition, the hierarchical structure consisting of the gel scaffold and pores with different size is visible, suggesting that the water removal process does not lead to a substantial collapse of the gel matrix. X-ray dispersive spectroscopy (EDS) measurements are conducted to analyze the elements in the CsBr-incorporated gel. Cs and Br with atomic ratios of 1:1 are evenly distributed at the gel surface (Fig. S2). The pore structures are beneficial to the PbBr_2_ solution penetration, and the homogeneously distributed CsBr grains ensure a sufficient reaction with PbBr_2_ to form PVSKs in the next step. Figure 1C shows the PVSK-gel sample featuring hierarchical structures. The sheet-like PVSKs are stacked to form large-scaled flower-like structures, and these superstructures are homogeneously distributed inside the whole gel (Fig. S3A).

As the solvent has a strong impact on the formation of PVSK crystals, other organic solvents, like 2-methoxyethanol (2-Me) and DMSO, are used for preparing PbBr_2_ solutions. The morphologies of the PVSK-gel composites fabricated by different solvents are displayed in Fig. S4A and B. For the 2-Me-based composite, the sample surface is covered with PVSK grains, which hinders the PbBr_2_ solution from penetrating into the gel inner. The cross-sectional SEM image further confirms that the PVSK grains only agglomerate at the sample surface to form a dense layer (Fig. S3B), which suggests that the PVSK crystallization only occurs at the sample surface. In contrast, different sized grains are observed in the DMSO-based sample (Fig. S4B). Additionally, the cross-section of the gel is full of inadequately reacted PVSK precursors (Fig. S3C). The morphology difference originates from the specific functional groups of organic solvents, which affect the coordination effect between PVSK precursors and solvents [34]. The coordination capacity of solvent with solute can be determined by the donor number (*D*_N_), where a higher *D*_N_ represents the ability to form a stronger coordination intermediate adduct with electron acceptor Pb^2+^. Referring to the literature, the *D*_N_ values of 2-Me, dimethylacetamide (DMAc), and DMSO solvents were determined to be 19.7, 27.8, and 29.8 kcal/mol, respectively [35,36]. The interaction between the solvents and PVSK precursors is studied by Fourier transform infrared (FTIR) measurements. As shown in Fig. S5A, the characteristic absorption peak of the C=O bond in DMAc shifts to lower wave number in the PbBr_2_ solution and then returns to the original position in the PVSK precursor solution [7]. The shift is ascribed to the C=O···Pb^2+^ coordination, and this coordination can be destroyed by CsBr [16]. Compared to the DMAc, no shift is observed in the characteristic peaks of 2-Me regardless of the PbBr_2_ and subsequent CsBr addition (Fig. S5B), indicating weaker coordination effect between Pb^2+^ and 2-Me as the solvent has less *D*_N_ [37]. The weak solute–solvent interaction causes a rapid reaction between PbBr_2_ and CsBr, leading to the formation of massive PVSK grains on the sample surface. For the DMSO, a new absorption peak located at 1,013 cm^−1^ appears in the PbBr_2_ solution (Fig. S5C), which originates from the PbBr_2_·DMSO intermediate adducts [38,39]. In the PVSK precursor solution, the peak intensity decreases steadily over time, indicating a slow reaction between PbBr_2_ and CsBr. This observation is visually verified by the color change after adding CsBr into the PbBr_2_ solutions with different solvents. As shown in Fig. S6, CsBr quickly turns into orange upon contact with the 2-Me-based solution and then the color remains unchanged over time. For the DMAc-based solution, CsBr gradually changes its color to orange with time, whereas it keeps the original color in the DMSO-based solution regardless of the storage time. This occurrence experimentally confirms that the coordination effect between the solvent and PVSK precursors plays a critical role in the crystallization rate of PVSK. Consequently, the obtained PVSK-gel samples based on these 3 solvents feature different luminescent looks under UV illumination (Fig. S7). The difference in the luminous intensity and luminescence uniformity is clearly visible for these 3 samples. For a quantitative comparison, the PLQY of the DMAc-based sample is determined to be 43.2%, which is superior to samples prepared with 2-Me and DMSO solvents, reading 30.9% and 17.7%, respectively.

Since DMAc is the optimized solvent for preparing the PVSK-gel composites, the following discussions will only focus on the samples with DMAc as the solvent. According to the x-ray diffraction (XRD) pattern (Fig. 1D), the measured peaks in PVSK-gel are assigned to Cs_4_PbBr_6_ PVSK phase. In detail, the diffraction peaks at 12.64°, 12.89°, 20.08°, 22.41°, 25.43°, 27.51°, 28.60°, 30.27°, and 38.99° are identified to be (012), (110), (113), (300), (024), (131), (214), (223), and (324) planes, respectively. EDS mapping shows that the Cs, Pb, and Br elements distribute homogeneously in the flower-like structures, and they have a molar ratio of about 4:1:6 (Fig. 1E and Fig. S8). This value is consistent with the ideal stoichiometric ratio of Cs_4_PbBr_6_, further confirming the crystal phase. Furthermore, the crystal structure is analyzed by high-resolution transmission electron microscopy (HRTEM) measurements (Fig. 1F). A *d*-spacing of 7.00 Å is visible, which corresponds to the (012) plane of Cs_4_PbBr_6_ PVSKs. In addition, the crystal is found to be covered with a polymer shell, which is in line with the SEM result that crystals are embedded in the gel matrix. Moreover, the TEM-EDS measurements show that the atomic ratio of Cs/Pb/Br in this grain agrees well with the element molar ratio of Cs_4_PbBr_6_ as well (Fig. S9). The photoluminescence (PL) spectrum in Fig. 1G demonstrates an emission peak at 520 nm with a narrow full width at half maxima (FWHM) of 22 nm for the PVSK-gel sample, and the green emission with color coordinate of (0.10, 0.77) is collected (inset of Fig. 1G). Regarding the long-term stability, it is found that the PL peak retains ~90% of its initial value for the PVSK-gel composite after storage at ambient conditions for 180 d (Fig. S10), demonstrating that the polymer shell stabilizes the luminescent performance of the PVSK grains. The photogenerated charge carrier lifetimes are investigated with time-resolved PL (TRPL) measurements (Fig. S11). From data modeling, the average PL lifetime (*τ*_ave_) of the PVSK-gel sample is 90.91 ns, which is similar with the values reported in the literature [40–42].

### Mechanism of CsPbBr_3_–Cs_4_PbBr_6_ phase transition

**Fig. 2.**
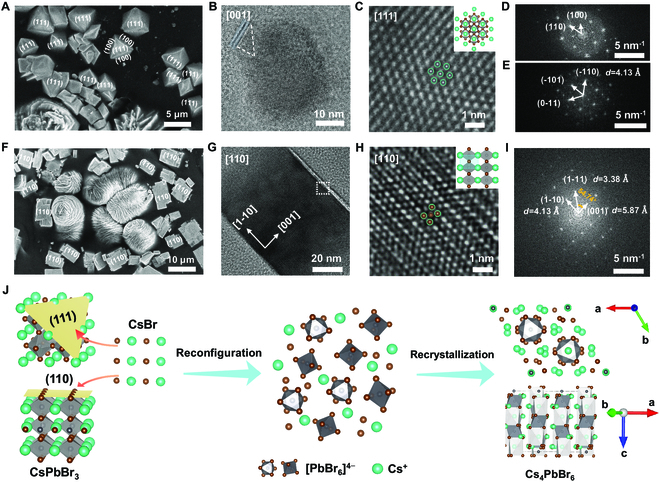
Structure evolution of PVSK crystals in the gel. (A) SEM image of the PVSK-gel composite with reaction time of 1 h. (B) TEM and (C) HRTEM with atomic-level images of CsPbBr_3_ inside the PVSK-gel (1 h). The inset in (C) is a schematic diagram of the atomic arrangement observed along the [111] zone axis in CsPbBr_3_. The green, gray, and brown spheres represent the Cs, Pb, and Br atoms, respectively. (D and E) Corresponding FFT patterns of the lattice areas in (B) and (C), respectively. (F) SEM image of the PVSK-gel with reaction time of 6 h. (G) TEM image of CsPbBr_3_ inside the PVSK-gel (6 h). (H) Zoom into the region with a dashed framework in (G). The inset is a schematic diagram of the atomic arrangement observed along the [110] zone axis. (I) Corresponding FFT pattern of the lattice area in (H). (J) Schematic diagram to depict the phase transition from CsPbBr_3_ to Cs_4_PbBr_6_ PVSKs in the Cs-rich condition.

In order to figure out the formation of the flower-like structures with Cs_4_PbBr_6_ phase, the samples are investigated by controlling the reaction time between the PbBr_2_ solution and CsBr-incorporated gel. The photographs of the PVSK-gels with reaction times of 1, 6, and 12 h are displayed in Fig. S12A to C, in which the green emission areas increasingly homogenize with increasing the reaction time. For SEM and wide-angle x-ray scattering (WAXS) characterizations, it is worth noting that the thickness of the PVSK-gel sample halves. To study this occurrence, SEM measurements are performed to examine these samples. For the reaction time of 1 h, the crystals have different morphology and size (Fig. S13). For the crystals with {100} facets exposed, they are truncated to have the exposure of the (111) plane with a triangular shape, and the flower-like structures with Cs_4_PbBr_6_ phase are located around them (Fig. 2A). In order to explore the fine crystalline structures, HRTEM measurements are carried out on a truncated cube. From the atomic arrangement (Fig. 2B) coupled with its corresponding fast Fourier transform (FFT) pattern (Fig. 2D), the [001] zone axis and the interplanar spacing of 5.87 and 4.13 Å for the (100) and (110) planes, respectively, are visible. Thus, the CsPbBr_3_ single crystal in Fig. 2B is identified with {100} facets exposed. By rotating the crystal with an angle of 45°, the truncation plane is examined as well (Fig. 2C). It is noted that the atomic-scale lattice matches well with the atomic arrangement of the (111) plane of CsPbBr_3_ (the inset of Fig. 2C). The central atoms and surrounding hexagon vertices correspond to the alternating Cs and Pb elements (green and gray overlapping spheres) with strong scattering properties [43]. In addition, the related FFT pattern in Fig. 2E further reveals the {110} crystal facets with a lattice spacing of 4.13 Å along the [111] zone axis. Furthermore, Fig. 2B demonstrates the ablation process of the CsPbBr_3_ crystal from one of its corners. In the upper-left corner of the cube, it is observed that the PVSKs are peeled off layer by layer along the (111) plane normal. Then, the exfoliated CsPbBr_3_ slices react with CsBr to form the Cs_4_PbBr_6_ stacks, which assemble together to get the flower-like structures.

When the reaction time reaches 6 h, the SEM image shows that the flower-like Cs_4_PbBr_6_ PVSKs are visible together with tetragonal CsPbBr_3_ crystals instead of the truncated cubes (Fig. 2F). The elemental ratios of both PVSKs are confirmed by SEM-EDS in Fig. S14. It is speculated that all truncated cubes convert to Cs_4_PbBr_6_ as more flower-like structures are present at this stage. The CsPbBr_3_ cuboids are investigated with HRTEM as well. Figure 2G shows that the exposed facet in this cuboid crystal is the (110) plane rather than the (100) plane (the truncated cube) [44]. The high-magnification image further verifies this finding (Fig. 2H), in which the atomic-level structure is consistent with the atomic arrangement observed along the [110] direction (inset of Fig. 2H). In a unit cell, the 4 brightest points on the corners (green and brown overlapping spheres) contain alternating Cs and Br atoms, and the brown sphere in the center is assigned to Br. The corresponding FFT pattern (Fig. 2I) well supports the structure analysis, which clearly identifies the presence of (100), (110), and (111) planes in the CsPbBr_3_ crystal based on the lattice *d*-spacing and angles. In addition, the high-magnification SEM image displays some white spots on the (110) plane (Fig. S15A), and the corresponding EDS spectra show that the Cs/Pb ratio in the white spot is greater than that in other regions. It is inferred that the reaction between CsBr and CsPbBr_3_ occurs in the white spot. Different from the grains with the exposed {100} facets, this kind of crystal reacts with CsBr at its (110) plane.

On the basis of the temporal analysis on the PVSK crystals, we find that 2 kinds of CsPbBr_3_ crystals are formed after immersing the CsBr-incorporated gel into the PbBr_2_ solution, one with {100} facets exposed and the other one with {110} facets exposed. Due to the high activity of the (111) plane in the former crystal species, CsBr tends to react with CsPbBr_3_ here, leading to layer-by-layer exfoliation of the CsPbBr_3_ PVSK along this normal plane. After reconfiguration and recrystallization, the layered Cs_4_PbBr_6_ stacks assemble into flower-like structures. After complete assumption of the truncated grains, the latter crystal kind still exists as the (110) plane is less active than the (111) plane. Finally, all CsPbBr_3_ PVSKs convert into the Cs_4_PbBr_6_ phase, since only flower-like structures are observed in the sample (Fig. 1C). For a clear illustration, the CsPbBr_3_–Cs_4_PbBr_6_ phase transition process is schematically shown in Fig. 2J.

**Fig. 3.**
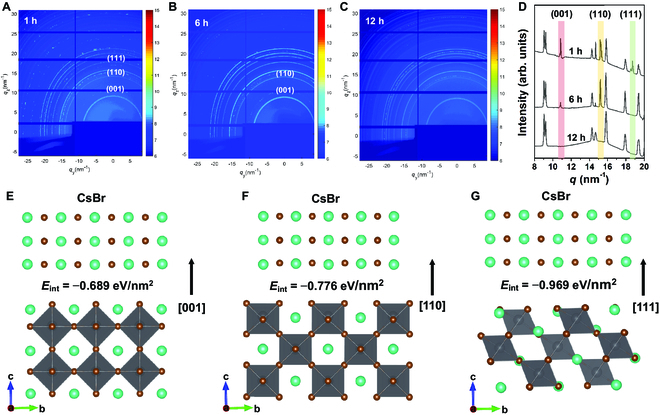
Interaction between CsBr and CsPbBr_3_ over time. (A to C) WAXS data and (D) the corresponding sector-averaged integrals of the PVSK-gels with reaction times of 1, 6, and 12 h, respectively. (E to G) DFT computational model of the interaction energies (*E*_int_) between CsBr with (001), (110), and (111) facets of CsPbBr_3_.

To gain more insights about the PVSK crystallization over time, WAXS and XRD measurements are performed on the PVSK-gel sample. Figure 3A to C shows the 2D WAXS data for different reaction time. The low Miller-index planes of CsPbBr_3_ including (001), (110), and (111) are indicated in the 2D WAXS data, and other Debye rings are identified as the crystal planes of Cs_4_PbBr_6_. The coexistence of CsPbBr_3_ and Cs_4_PbBr_6_ is both found in the reaction time of 1 and 6 h, whereas only Cs_4_PbBr_6_ is observed after 12 h. Figure 3D depicts the sector-averaged integrals of the WAXS data. It can be seen that all low Miller-index facets of CsPbBr_3_ exist in the early stage (the reaction time of 1 h), and the (111) plane (*q* = 18.6 nm^−1^) is clearly seen, which is in line with the SEM observation of the truncated cubes enclosed by {001} facets (*q* = 10.7 nm^−1^) in Fig. 2A. For the reaction time of 6 h, the signal of the (111) plane disappears and the relative intensity of the (001) plane decreases as a consequence of the complete consumption of truncated cubes. However, the intensity ratio of (110)/(001) planes largely increases compared to the sample with 1-h reaction time, which is due to only CsPbBr_3_ crystals with {110} facets exposed (*q* = 15.2 nm^−1^) remaining. Along with the reaction time, CsPbBr_3_ continues to interact with CsBr until it completely converts into Cs_4_PbBr_6_, which is manifested in the absence of CsPbBr_3_ diffraction signals after 12 h.

In order to understand the correlation between exposed facets and phase transition, density functional theory (DFT) studies are conducted to calculate the interaction energies (*E*_int_) between CsBr and (001), (110), and (111) planes of CsPbBr_3_. As shown in Fig. 3E to G, CsBr exhibits the lowest *E*_int_ of −0.969 eV/nm^2^ with CsPbBr_3_ (111) plane, while this value increases to −0.776 and −0.689 eV/nm^2^ for the (110) and (001) planes, respectively. The *E*_int_ difference suggests that the priority of the interaction between CsBr and CsPbBr_3_ facets follows (111) > (110) > (001). Therefore, the (111) facet is the most sensitive to CsBr, which is manifested in the observation of the exposed (111) plane after 1-h reaction time. Since the (110) facet has a higher *E*_int_, the related crystals still exist after 6 h. The (001) facet with the most bonded atoms has the largest *E*_int_; thus, CsBr tends to react at the (111) plane rather than this facet [45–47].

XRD patterns in Fig. S12D confirm the coexistence of CsPbBr_3_ and Cs_4_PbBr_6_ and the corresponding phase transition in the PVSK-gel sample as well. It can be seen that the characteristic peaks of CsPbBr_3_ decrease in intensities (orange highlights), while those of Cs_4_PbBr_6_ increase (purple highlights) over time. This occurrence further confirms that CsPbBr_3_ gradually converts into Cs_4_PbBr_6_.

### Demonstration of PVSK-gels with full-color emission

**Fig. 4.**
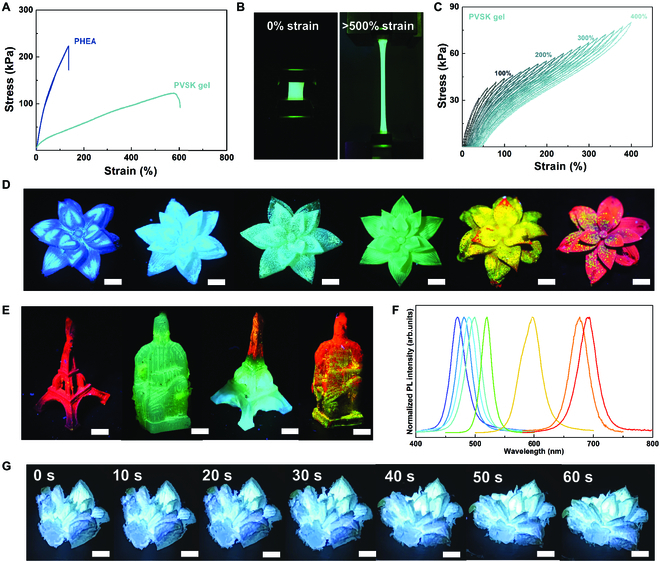
Mechanical properties and 3D printing implementation. (A) Tensile stress-strain curves for the PHEA and PVSK-gel samples. (B) Stretchable PVSK-gel sample under UV irradiation. (C) Successive tensile cycling tests of the PVSK-gel with corresponding strains. (D) Demonstrations of 3D printing stereoscopic flowers with blue-to-red emission colors under UV illumination. Exemplary illustration of Eiffel Tower and Terracotta Warriors with (E) single-color and full-color luminescence under UV excitation. (F) Normalized PL spectra of different PVSK-gel samples. (G) Shape-memory demonstration of the PVSK-gel composites. Scale bar, 3 mm.

The tensile measurements are employed to study the mechanical property of the PVSK-gel sample. For comparison, PHEA is tested as well. As depicted in tensile stress–strain curves (Fig. 4A), PHEA exhibits the rigidity with an ultimate tensile strength (UTS) of 223.1 kPa and the elongation at a break (*λ*) of 135.5%. After the PVSK nanocrystal incorporation, the flexibility is significantly enhanced for the composite, which is manifested in a decrease of UTS to 122.5 kPa and an increase of λ to 578.1%. For comparison, the pure gel is prepared with the same procedures as the PVSK-gel sample but without the introduction of PVSKs. Its UTS decreases to 74.2 kPa and *λ* increases to 665.9% (Fig. S16). This is due to the fact that the hydrogel generally increases the distance between the polymer molecular chain segments and reduces the chain entanglement (Fig. S17). In addition, the presence of PVSK crystals produces a stress concentration effect, yielding the resin matrix around the grains, consequently giving rise to a toughening effect. The PVSK-gel sample before and after stretching under UV illumination is demonstrated in Fig. 4B. It can be seen that the entire sample exhibits homogeneous green emission even at >500% strain. Excellent flexibility of the PVSK-gel composites provides strong mechanical support for PVSK grains in the gel matrix to stabilize their optoelectronic properties under the deformation state. In addition, the PVSK-gel sample is subjected to successive loading–unloading cycling test, with 20 cycles of arithmetic variation in the strain range from 20% to 400% (Fig. 4C). This composite maintains mechanical stability and presents small hysteresis loops, indicating a good elastomer property with excellent resilience ability at all set strains.

The molding of the PVSK-gel sample is mainly determined at the UV curing step; thus, it is possible to use digital light processing (DLP) 3D printing technology to fabricate 3D structures at the first step. In this work, digital UV spots are projected on the CsBr/HEA-monomer mixture to trigger the localized photopolymerization, followed by a layer-by-layer fabrication of 3D structures with customized geometry. Notably, 2 steps are used to prepare PVSK-gel composites instead of one-pot production in this work; thus, it is easy to regulate the precursor components and/or ratios individually in both steps to obtain different PVSKs after 3D architecture establishment, leading to tunable PL colors. Following this concept, a set of stereoscopic PVSK flowers with high-definition features are manufactured by a 3D printing technique, displaying a series of emission colors from blue, green, to red under 365-nm UV excitation (Fig. 4D). Furthermore, luminescent PVSK-gels in other complex shapes, such as Eiffel Tower and Terracotta Warriors, not only are manufactured successfully but also integrate multicolor emissions in the same device (Fig. 4E). This is simply achieved by altering components and concentrations of the lead halide solution. This demonstration shows that various emission colors can be artistically designed in local domains, giving a proof of practicability for the 3D printing PVSK models. Figure 4F depicts the normalized PL spectra of PVSK-gel samples, implying that the composition modulation of all-inorganic PVSKs can be effectively achieved inside the polymer carriers for full-color emissions. The detailed implementation parameters of the multicolor PVSK experiments are listed in Table S1. Moreover, as the flexible PVSK-gel composites allow for various forms of deformation due to their excellent mechanical property, the shape-memory function is possible after structural programming. As illustrated in Fig. 4G, the blooming flower prototype is programmed with closed petals at 25 °C, and then the temperature is cooled down to −40 °C to maintain the shape (Fig. S18). During the steady increase of the temperature to 25 °C, the flower gradually recovers its original form with blooming petals (Movie S1). In this dynamic experiment, the blue emission of this sample remains stable under 365-nm UV excitation.

These demonstrations prove that the 2-step process in this work is a universal technique for fabrication of the PVSK-gel composites with emissions over the full visible spectral range. Moreover, the compatibility with DLP 3D printing technology platform enables rapid prototyping and customized model manufacturing. The printed free-standing flexible devices with recoverability are able to fulfill diverse demands.

## Discussion

In summary, a 2-step route is implemented to incorporate all-inorganic PVSK crystals into gel matrix, which is demonstrated to be a universal approach to prepare free-standing PVSK-gel composites with full-color luminescence. By penetrating PbBr_2_ solution into the CsBr-incorporated hydrogel, the obtained PVSK-gel composite exhibits a PLQY value of 43.2% and a narrow PL FWHM of 22 nm, and retains ~90% of the PL performance after storage at ambient conditions for 180 d. The excellent optoelectronic property is closely related to the sample morphology. It is found that 2 forms of CsPbBr_3_ crystals are formed inside the gel matrices at the initial stage. Over time, all CsPbBr_3_ gradually react with the existing CsBr to form Cs_4_PbBr_6_ phase with a flower-like structure. Experimental and theoretical studies reveal that CsBr interacts preferentially with the (111) facet of CsPbBr_3_ compared to the (110) and (001) planes due to the lowest interaction energy. Furthermore, the PVSK-gel composite possesses outstanding elasticity, tensile strain (>500%), and shape-memory function, which enables the compatibility with DLP 3D printing technology. The customized stereoscopic devices are demonstrated to have different architectures with full-color emissions.

The developed strategy in this work solves the compatibility issues between different PVSK precursors and polymer carriers, enabling various PVSK-gel combinations based on the same manufacturing processes. This new idea shows great potential in manufacturing optoelectronic devices with multifunctional requirements and broad applications due to its strong technical versatility and facile processability.

## Materials and Methods

### Materials

Cesium bromide (CsBr, 99.99%), cesium iodide (CsI, 99.99%), lead chloride (PbCl_2_, 99.99%), and lead bromide (PbBr_2_, 99.99%) were purchased from TCI. Hydroxyethyl acrylate (HEA) and poly(ethylene glycol) diacrylate (PEGDA) were purchased from Adamas-β. Water-soluble lithium diphenyl (2,4,6-trimethylbenzoyl) phosphine oxide (TPO-Li) was synthesized according to the method reported by Wang et al. [48]. 2-Methosxyethanol (2-Me), DMAc, and DMSO were purchased from Adamas-β. All materials were used as received without further purification.

### Preparation of mixed solution

CsBr aqueous solution A was prepared by dissolving CsBr into deionized water at a concentration of 1 M and then stirred at room temperature for 10 min. Polymer monomer solution B was prepared by mixing 15 g of HEA (monomer), 0.15 g of PEGDA (crosslinker), and 0.15 g of TPO-Li (photo-initiator) at room temperature. Then, solution A, solution B, and deionized water were mixed with a volume ratio of 3:2:5 to finalize the solution for the first step.

### Preparation of CsBr-gels

The previously prepared solution (400 ml) was poured into a Teflon mold (1.5 cm × 2 cm × 1 mm) for UV photo-polymerization. After exposure for 30 min in a UV chamber, the obtained hydrogels were freeze-dried (FD) for 1 day to remove water completely. The white CsBr-incorporated gels were obtained. With respect to the samples used for WAXS and SEM characterizations, the gels are thinned to a thickness of 0.5 mm.

### Preparation of PVSK-gels

PbBr_2_ solution (0.05 M) with DMAc as the solvent was achieved by being stirred at 60 °C for 1 h. The CsBr-incorporated gel was immersed into the PbBr_2_-DMAc solution for 24 h and then annealed at 100 °C for 30 min to obtain the PVSK-gel composite. For the PVSK-gel samples prepared with 2-Me and DMSO, the preparation processes are the same except for different solvents of the PbBr_2_ solution.

### Preparation without PVSK-gels

The hydrogel is UV-cured after mixing deionized water and polymer monomer solution B at a volume ratio of 8:2. After FD procedure, the gel is immersed into DMAc solvent, followed by the annealing process.

### Preparation of PHEA

PHEA is directly UV-polymerized from polymer monomer solution B without other subsequent processing steps.

### Characterizations

Field-emission SEM micrographs were acquired on an FEI Verios G4 instrument at 10 kV. FLS1000 from Edinburgh Instruments was carried out to test steady-state and time-resolved PL spectra, with excitation wavelengths of 365 and 380 nm, respectively. Absolute PLQYs were measured on C9920-02 system (Hamamatsu Photonics K. K., Japan) at 365-nm excitation. X-ray diffractometer characterization was performed using a Bruker AXS D8 instrument equipped with Cu-Kα radiation source to acquire the PVSK crystal structures. The microstructures of PVSK were measured by HRTEM instrument (FEI Talos F200X) equipped with a high-angle annular dark field (HAADF) detector. WAXS measurements were carried out at the BL16B1 beamline of the Shanghai Synchrotron Radiation Facility (SSRF) using an x-ray with an energy of 10 keV and a wavelength of 1.24 Å. The sample-to-detector distance and beam center were calibrated by standard sample, i.e., silver behenate (AgB). The tensile and successive tensile cycling tests were performed on a universal tension tester (INSTRON, 3344, 2 kN, USA), where the stretching speed is 5 mm/1 min. A DLP 3D printer (Thorlabs, NJ, USA) equipped with 405-nm light source projector was used in this work. The constructed 3D models are sliced into series of 2D images through computer program, followed by modulating into patterned UV light by digital micro-display (DMD). The stereo structures are printed layer by layer, and the exposure time of each layer is set to 0.8 s through the custom. During the printing process, it is permitted to pause and change the printing solution to manufacture multicolor PVSK-gels.

### Computational analysis

We performed the first-principles calculations in the frame of DFT with the program package CASTE, using the plane-wave ultra-soft pseudopotential (PW-USPP) method and the Perdew–Burke–Ernzerhof (PBE) form of generalized gradient approximation (GGA) exchange-correlation energy functional. The structure optimization for (100), (110), and (111) planes of CsPbBr_3_ covered by CsBr (110) plane was carried out using means of the Broyden–Fletcher–Goldfarb–Shanno (BFGS) algorithm by allowing all atomic positions to be varied and fixing lattice parameters. They would stop until the total energies were converged to 10^−5^ eV/atom, the forces on each unconstrained atom were smaller than 0.03 eV/Å, the stresses were lower than 0.05 GPa, and the displacements were less than 0.001 Å. The plane-wave cutoff was chosen to 340 eV. The *k*-point mesh of 1 × 1 × 1 was used for Brillouin zone (BZ) sampling for all slabs.

This slab was separated by a 15-Å vacuum layer in the *z* direction between the slab and its periodic images. During structural optimizations of the surface models, a 1 × 1 × 1 gamma-point centered *k*-point grid for BZ was used and the bottom 4 atomic layers of CsPbBr_3_ and bottom 3 atomic layers of CsBr were fixed, while other atoms were relaxed.

The interaction energy (*E*_int_) between (100), (110), and (111) planes of CsPbBr_3_ with CsBr (110) plane was defined as:

$$E_{\text{int}}=1/A\left(E_{\text{CsPbBr}3/\text{CsBr}}\;–\;E_{\text{CsPbBr3}}\;–\;E_{\text{CsBr}}\right)$$

where *E*_CsPbBr3/CsBr_, *E*_CsPbBr3_, and *E*_CsBr_ are the energies of interaction system. *A* is the interfacial area.

## Data Availability

All data needed to evaluate the conclusions in the paper are present in the paper and/or the Supplementary Materials.
