# Supplementary material for: A Universal Approach Toward Intrinsically Flexible All-Inorganic Perovskite-Gel Composites with Full-Color Luminescence
Source: Research (Wash D C). 2024 Jul 8;7:0412. doi: 10.34133/research.0412 (PMC11227898; doi:10.34133/research.0412)
Supplement: Supplementary 1 — Figs. S1 to S18 Table S1 Movie S1 [file research.0412.f1.zip › Research-supplementary_materials.docx]

Supplementary Materials for

**A Universal Approach Towards Intrinsically Flexible All-Inorganic-Perovskite-Gel Composites with Full-Color Luminescence**

Dourong Wang^1‡^, Jingjing Cui^1‡^, Yang Feng^1‡^, Yunlong Guo^1^, Jie Zhang^1^, Yaqi Bao^1^, Haoran Deng^1^, Ruiqian Chen^1^, Xinxin Kang^1^, Biao Zhang^1^*, Lin Song^1^*, Wei Huang^1,2,3^*

^1^ Frontiers Science Center for Flexible Electronics (FSCFE), Institute of Flexible Electronics (IFE), Ningbo Institute of Northwestern Polytechnical University, Northwestern Polytechnical University, 127 West Youyi Road, Xi'an 710072, China

^2^ Key Laboratory of Flexible Electronics (KLOFE) & Institute of Advanced Materials (IAM), Nanjing Tech University (NanjingTech), 30 South Puzhu Road, Nanjing, 211816 China.

^3^Key Laboratory for Organic Electronics & Information Displays (KLOEID) and Institute of Advanced Materials (IAM), Nanjing University of Posts & Telecommunications, Nanjing, 210023 China.

^*^Address correspondence to: [iambzhang@nwpu.edu.cn](mailto:iamb@nwpu.edu.cn) (B.Z.); [iamlsong@nwpu.edu.cn](mailto:iamlsong@nwpu.edu.cn) (L.S.); [vc@nwpu.edu.cn](mailto:vc@nwpu.edu.cn) (W.H.)

‡These authors contributed equally: Dourong Wang, Jingjing Cui, Yang Feng.


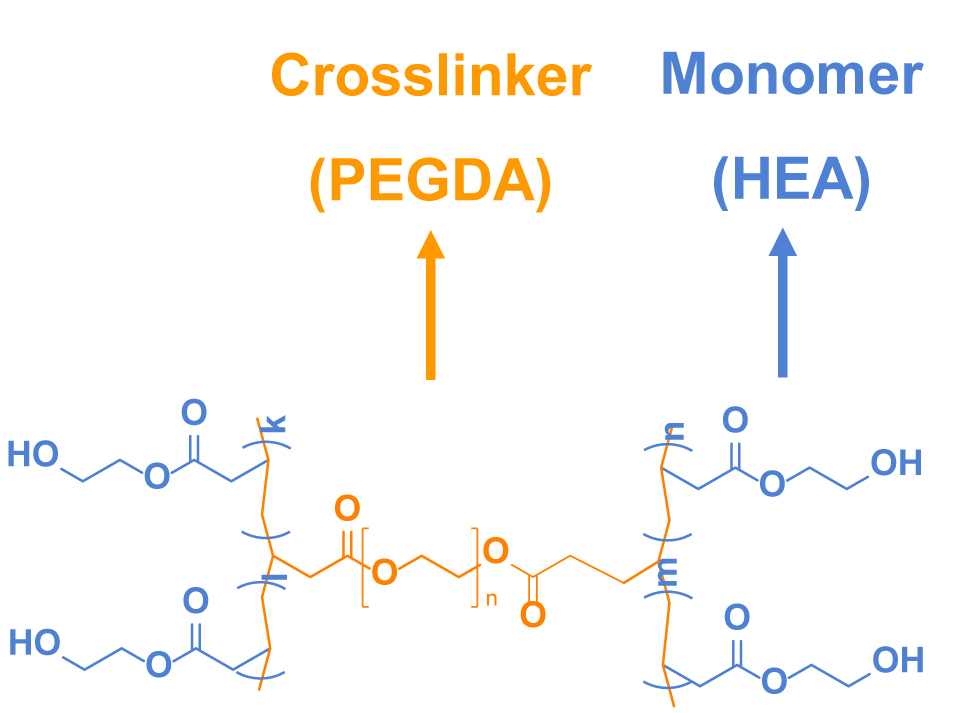


Fig. S1. Chemical structure of PHEA gel after UV curing.


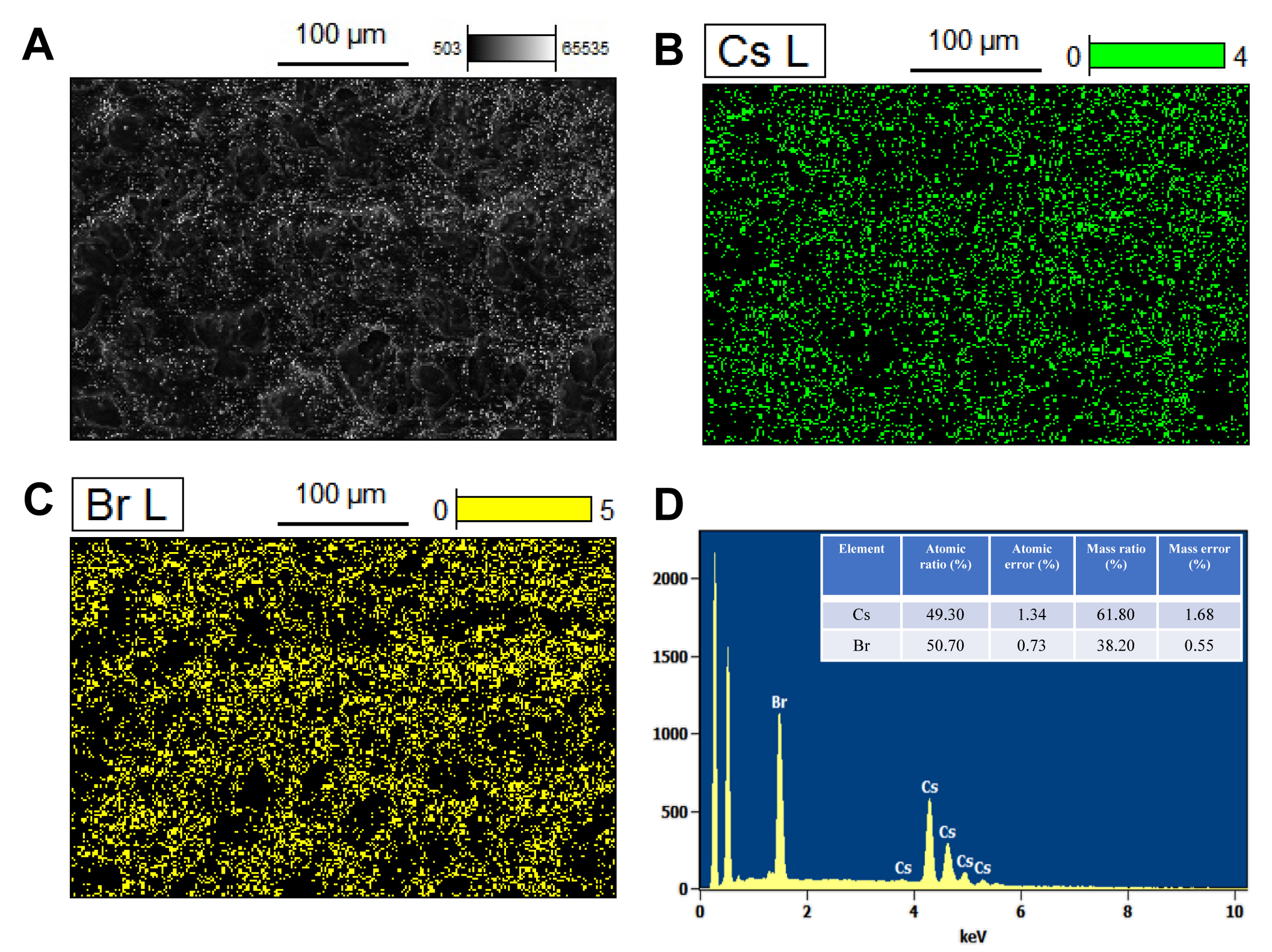


Fig. S2. (A) SEM image, (B, C) the corresponding EDS elemental maps and (D) the elemental analysis of the CsBr-incorporated hydrogel after water removal.


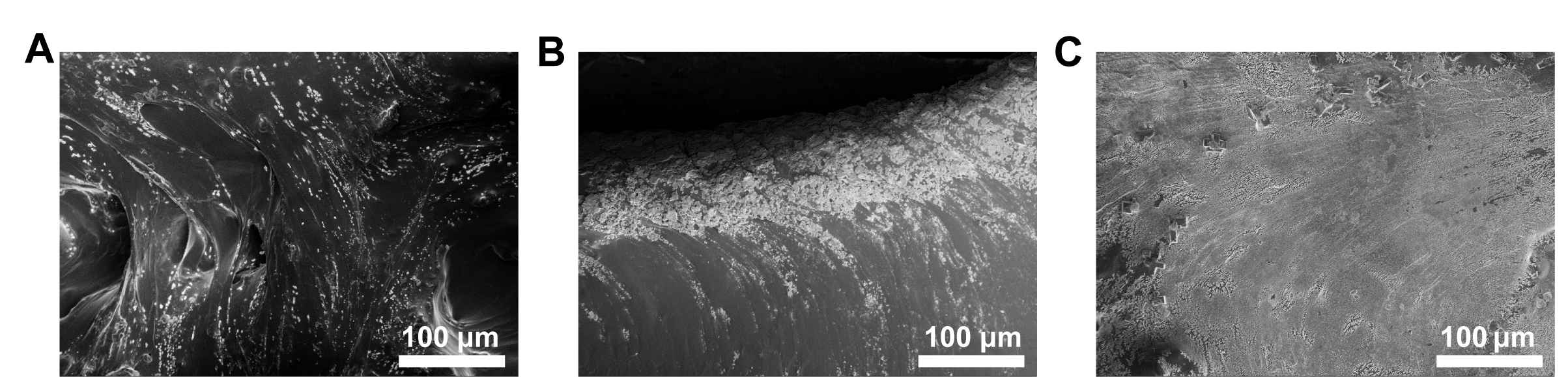


Fig. S3. Cross-sectional SEM images of the PVSK-gel composites prepared by PbBr_2_ solutions in (A) DMAc, (B) 2-Me and (C) DMSO.


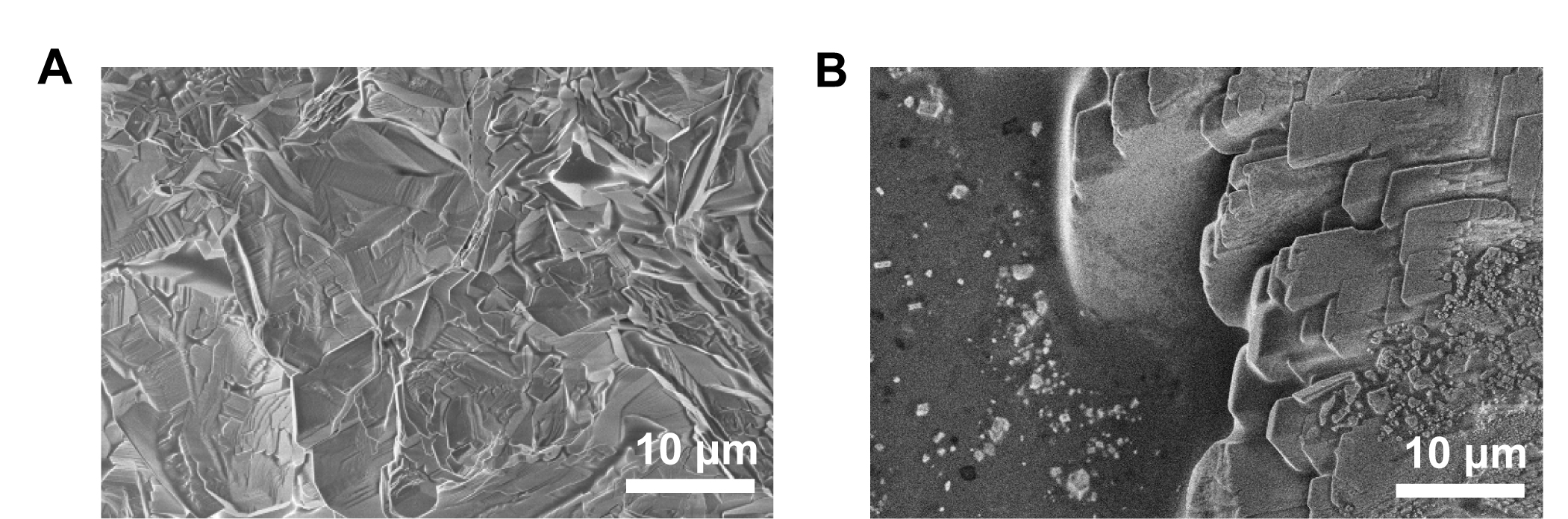


Fig. S4. SEM images of the PVSK-gel samples induced by PbBr_2_ solutions in (A) 2-Me and (B) DMSO solvents.


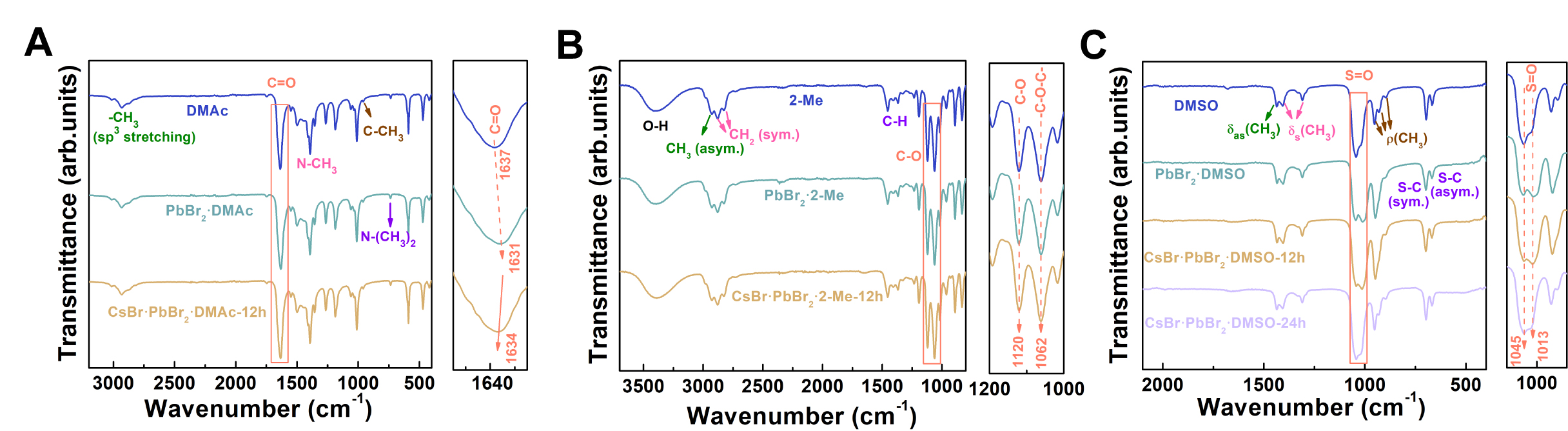


Fig. S5. FTIR spectra of perovskite precursor solutions with (A) DMAc, (B) 2-Me and (C) DMSO as solvents.


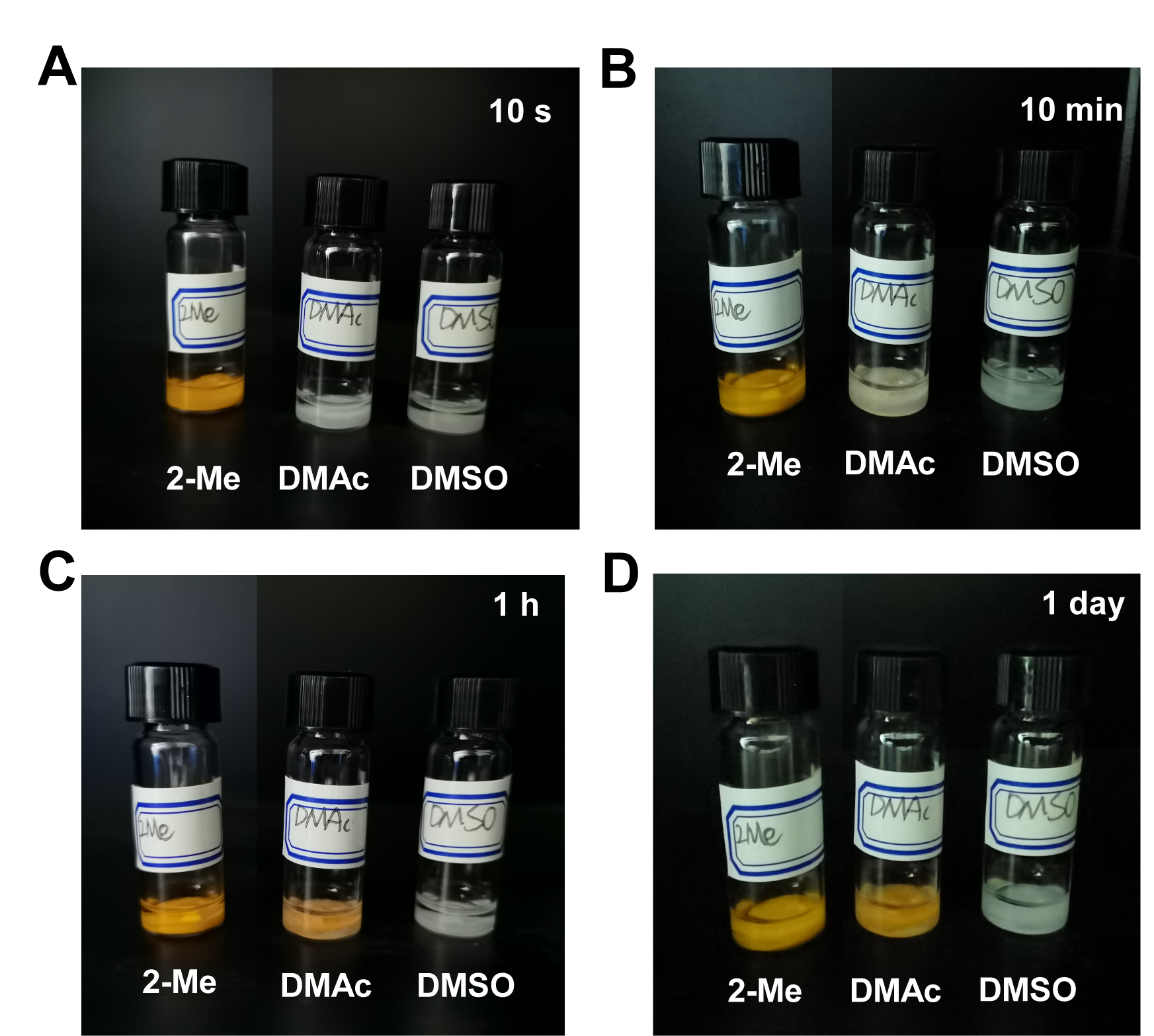


Fig. S6. Evolution process of CsBr powders in PbBr_2_ solutions with different solvents at the time as indicated.


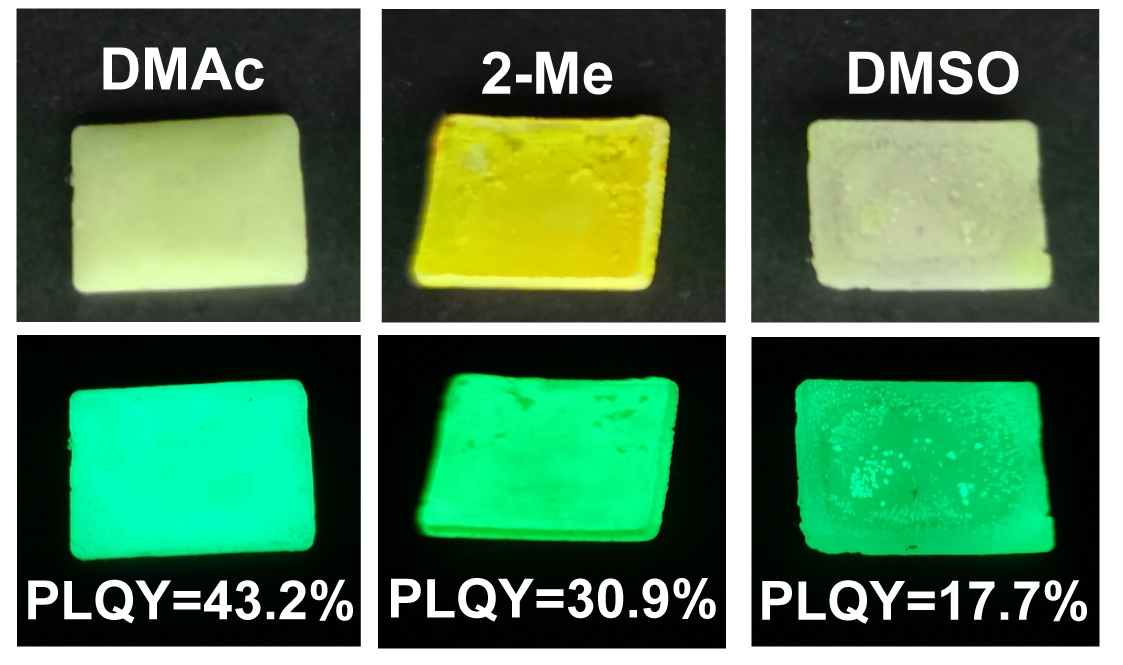


Fig. S7. Photographs of the PVSK-gel samples prepared with different solvents under the ambient light and UV light illumination.


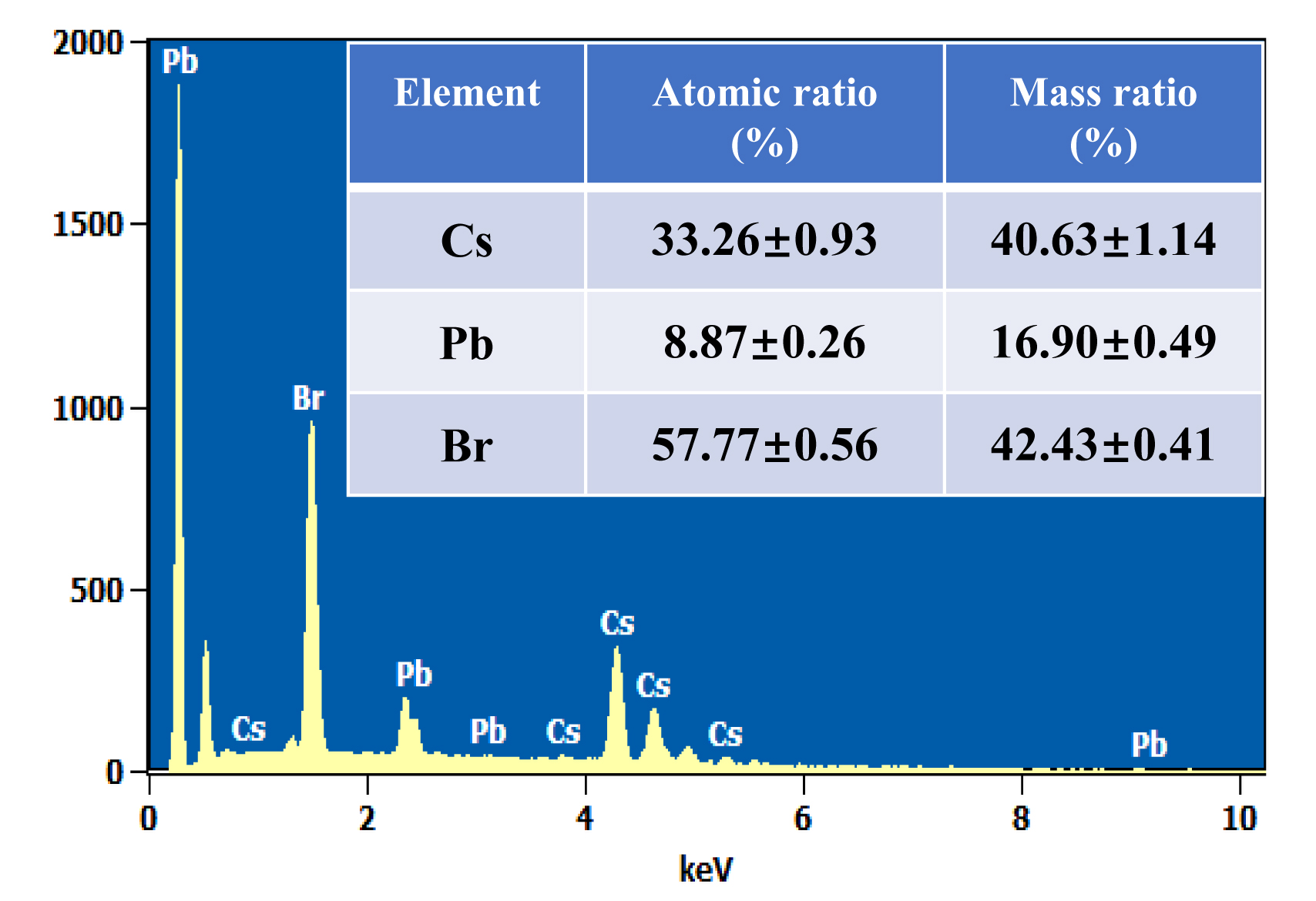


Fig. S8. The elemental analysis of the region marked with an orange square in Fig. 1E.


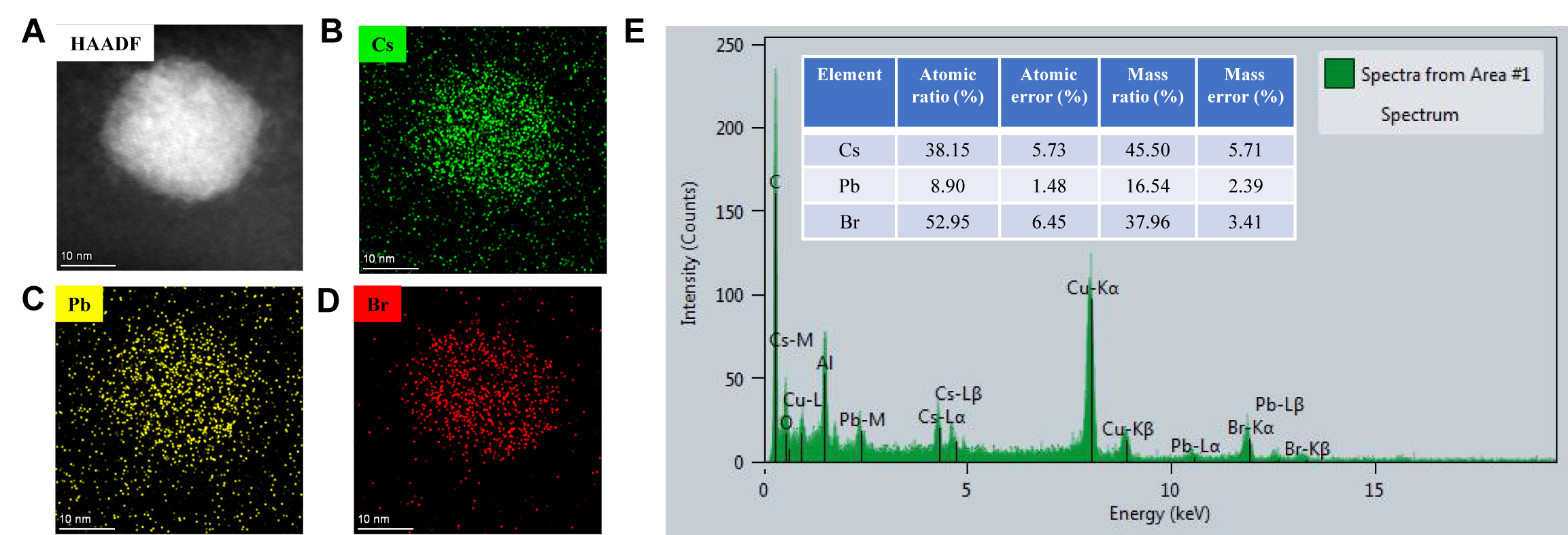


Fig. S9. (A) High-angle annular dark-field transmission electron microscopy (HAADF-TEM) image, (B-D) the corresponding EDS mapping images, and (E) the elemental analysis of Cs_4_PbBr_6_ inside the PVSK-gel matrix.


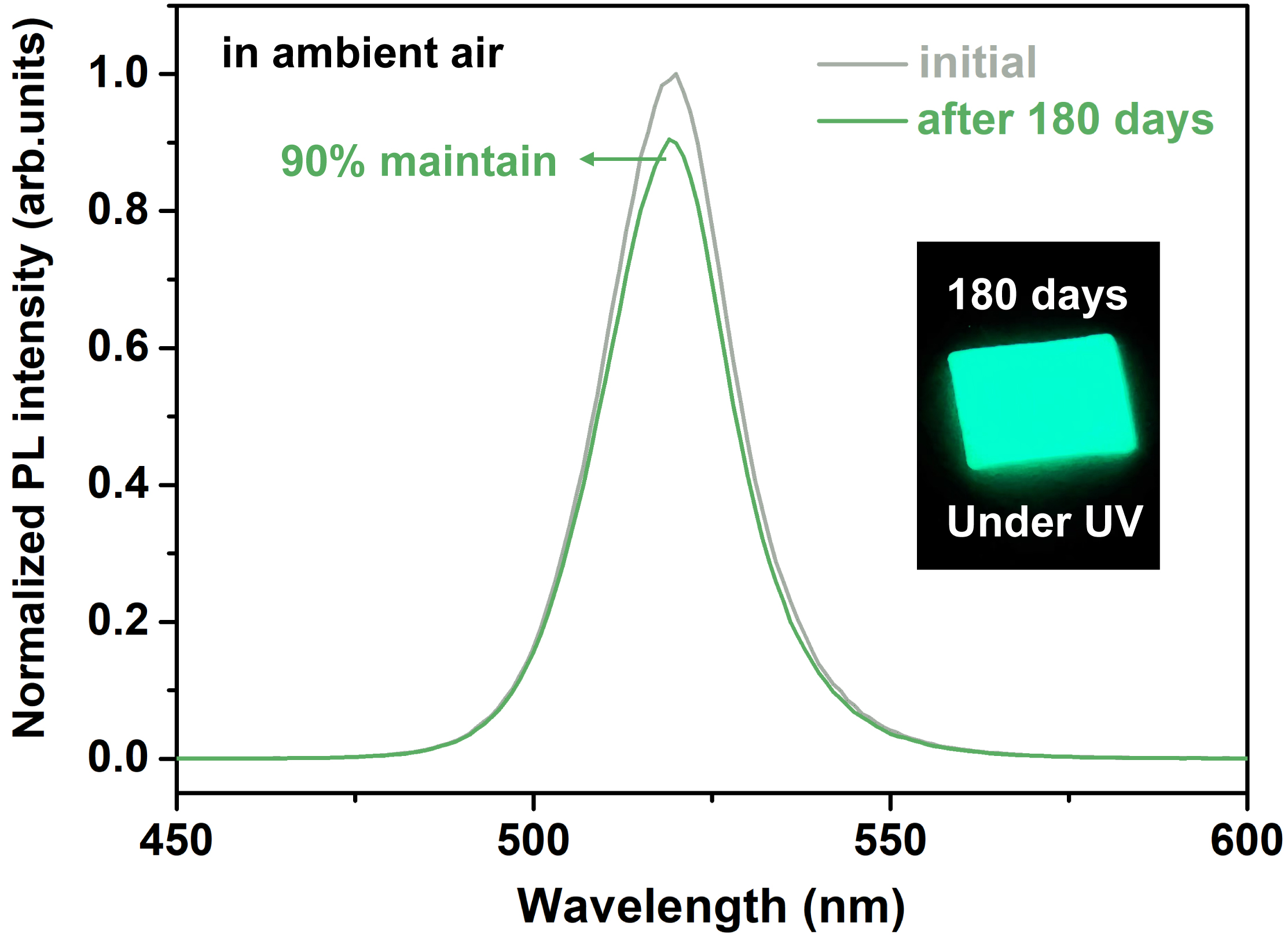


Fig. S10. Normalized PL spectra (λ_Excitation_=365 nm) of the PVSK-gel composites before and after storage at ambient conditions for 180 d (Inset: the luminescent photograph of the sample after storage at ambient conditions for 180 d).

**
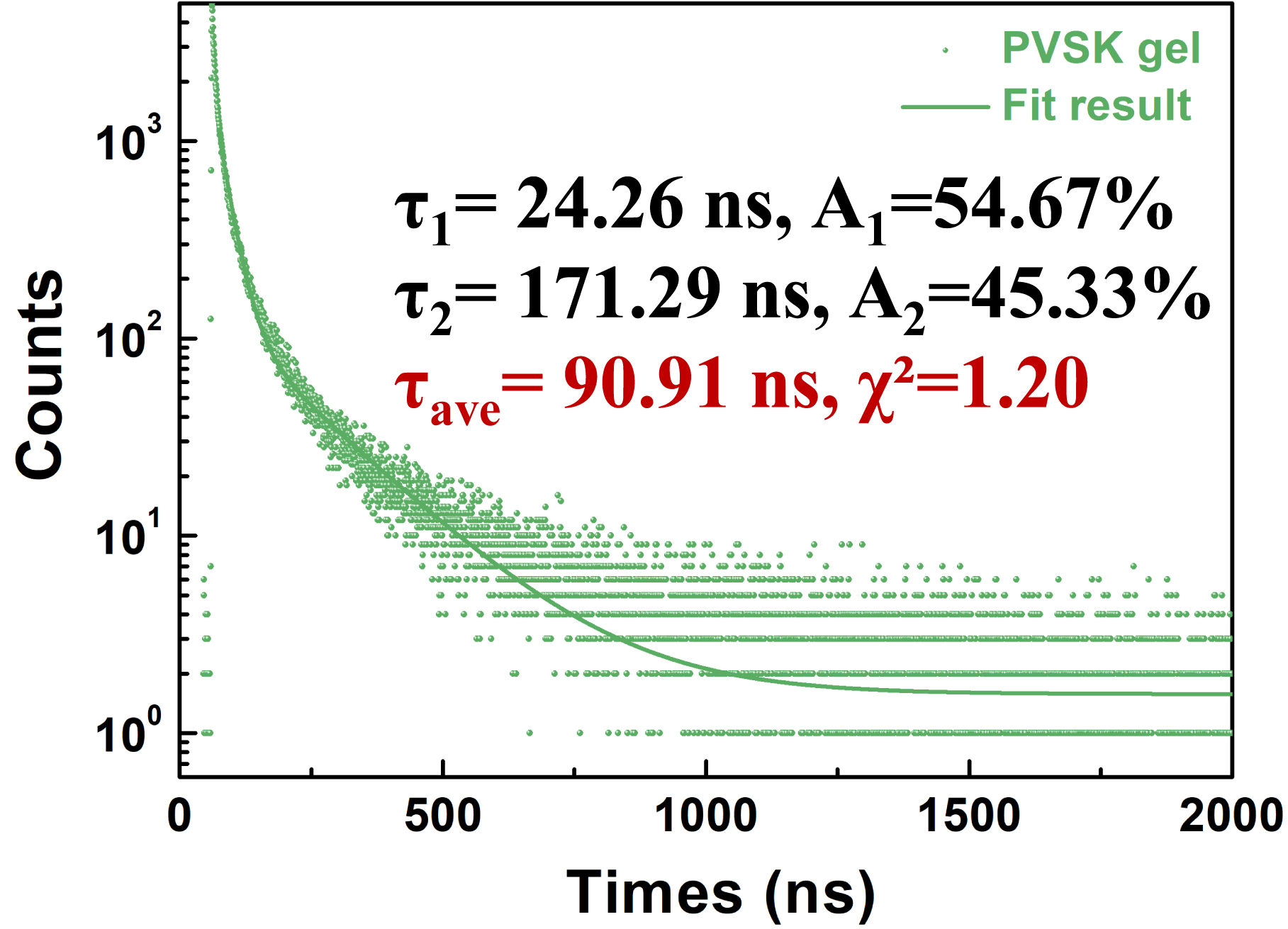
**

Fig. S11. Time-resolved PL (TRPL) spectra (λ_Excitation_=380 nm) of the PVSK-gel composite (green dots) fitted by a biexponential function (solid line).


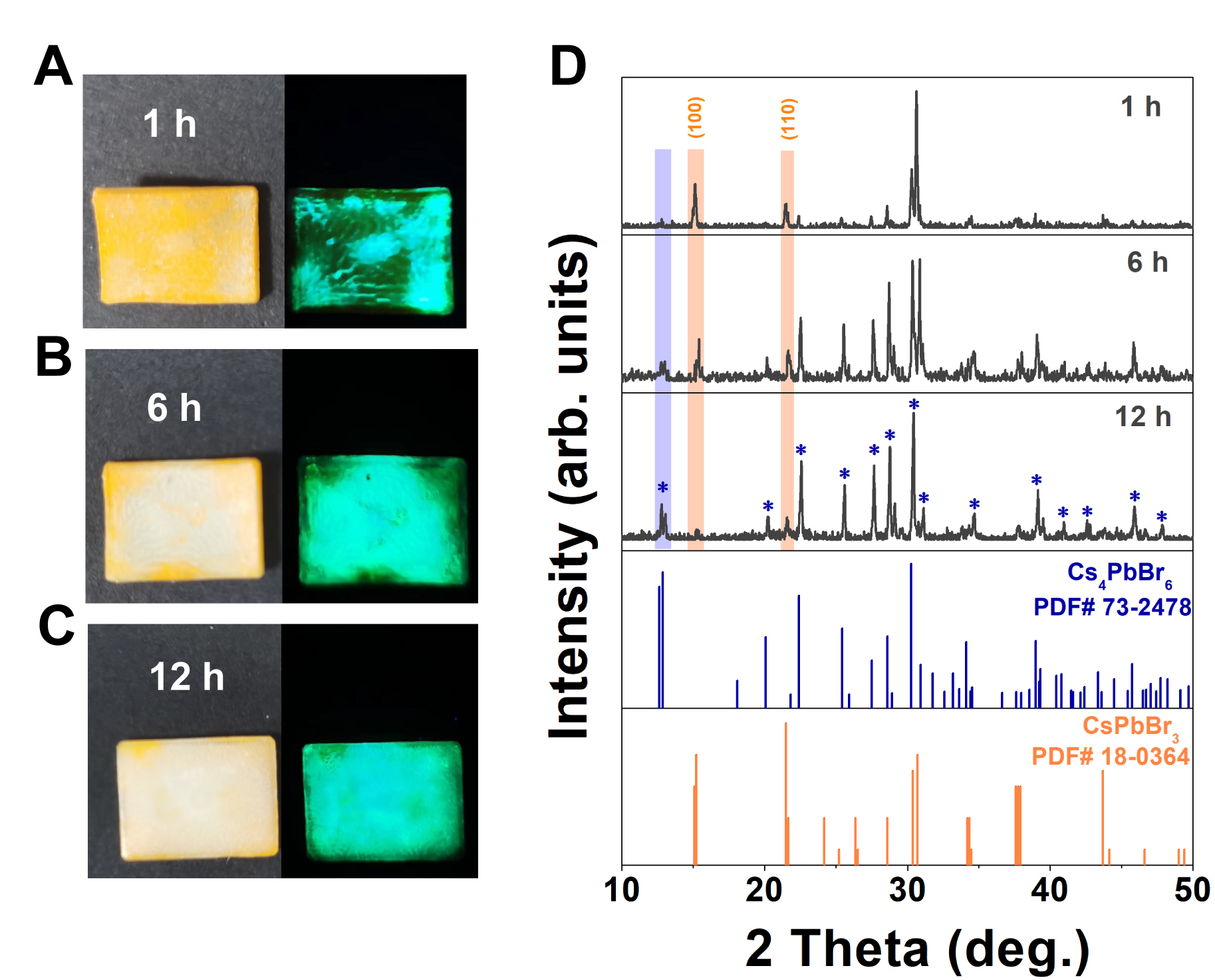


Fig. S12. Photographs of the PVSK-gel samples under the ambient light and UV light illumination with the reaction time of (A) 1 h, (B) 6 h and (C) 12 h. (D) The corresponding XRD curves. The vertical dark blue and orange sticks represent the peak positions and their respective relative intensities of Cs_4_PbBr_6_ and CsPbBr_3_, respectively.


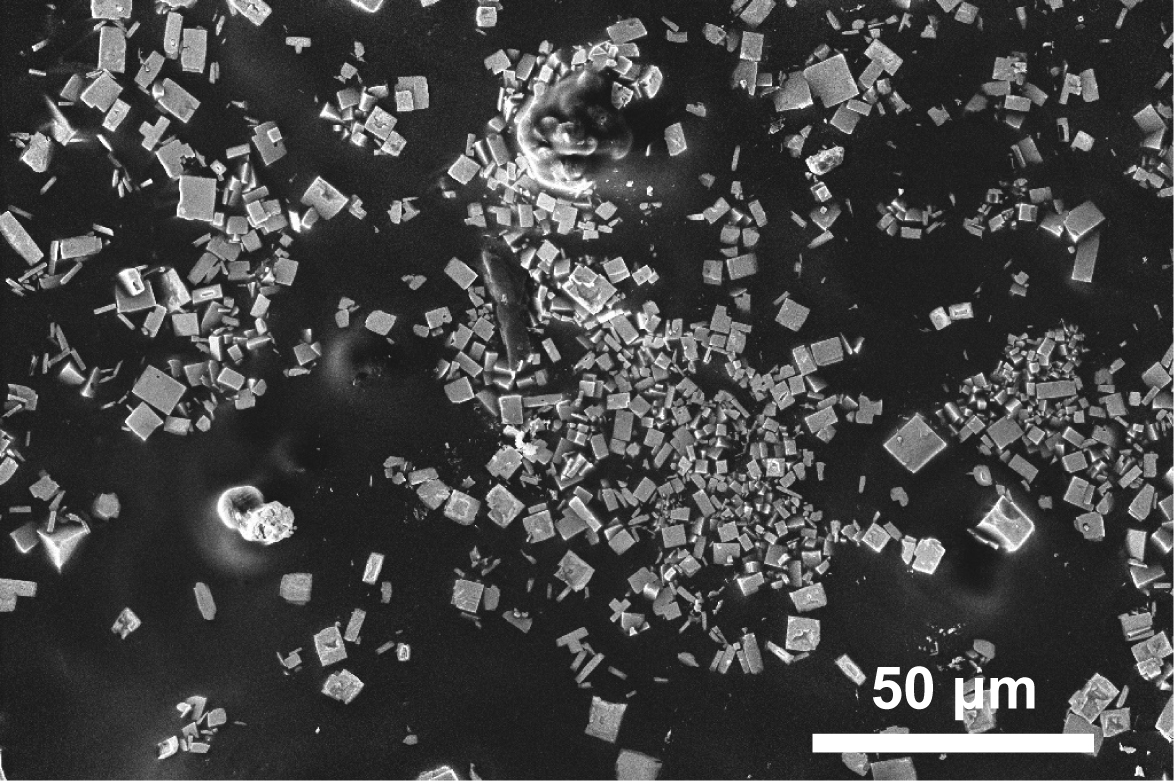


Fig. S13. SEM image of the PVSK-gel sample with reaction time of 1 h.


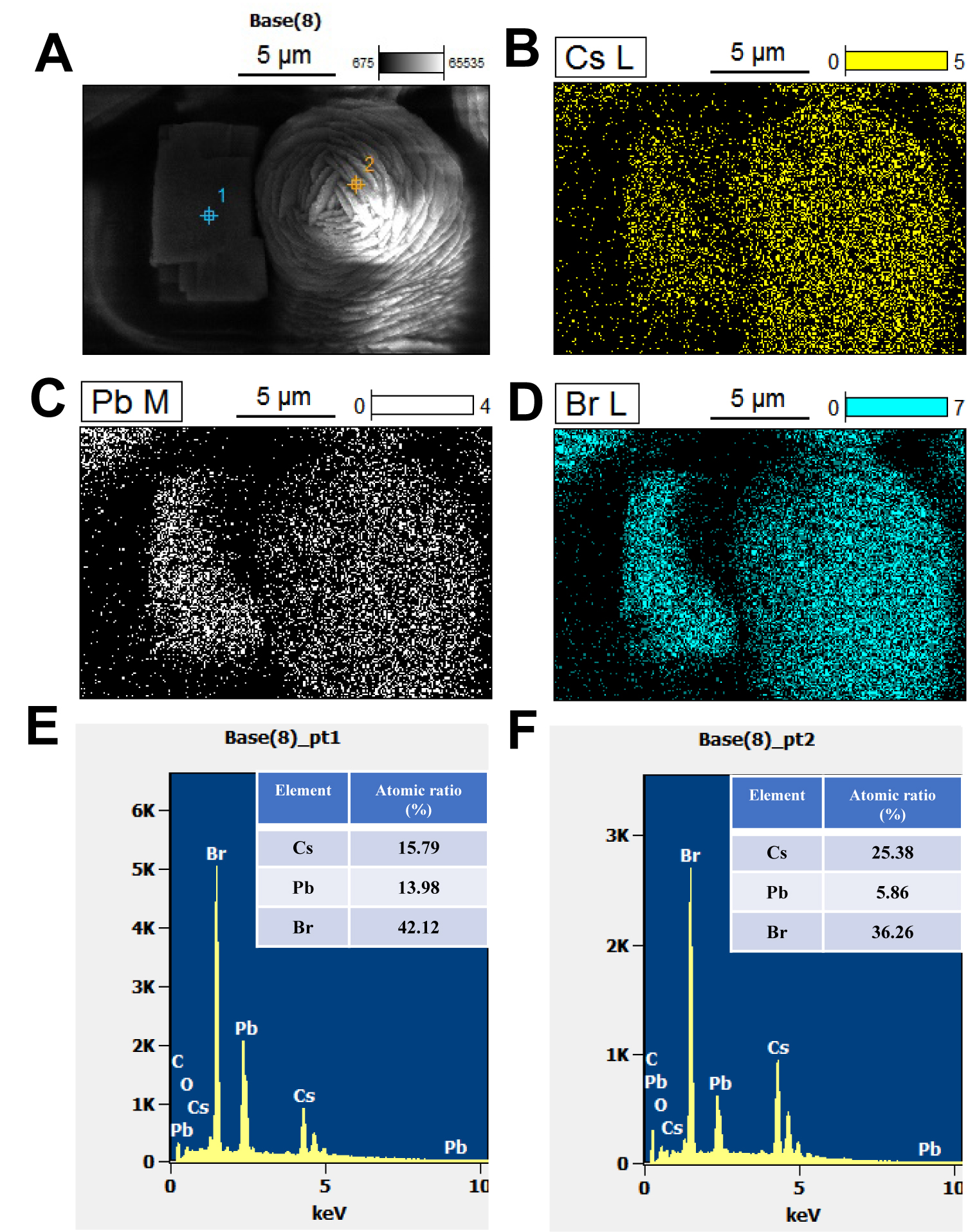


Fig. S14. (A) SEM image, (B-D) the corresponding EDS mapping images of the PVSK-gel sample with reaction time of 6 h. (E, F) Elemental analysis of the two spots marked in (A).


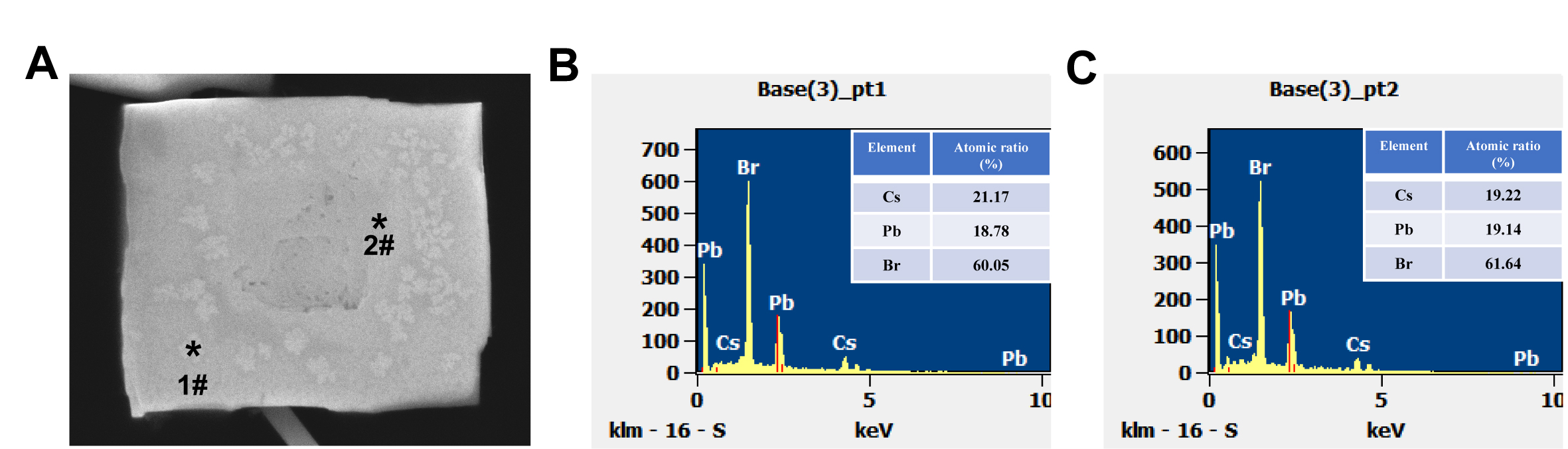


Fig. S15. (A) SEM image of the CsPbBr_3_ crystal with the {110} facets exposed. (B and C) Elemental analysis of the two spots marked in (A), respectively.





Fig. S16. Tensile stress-strain curves for the w/o PVSK gel sample.


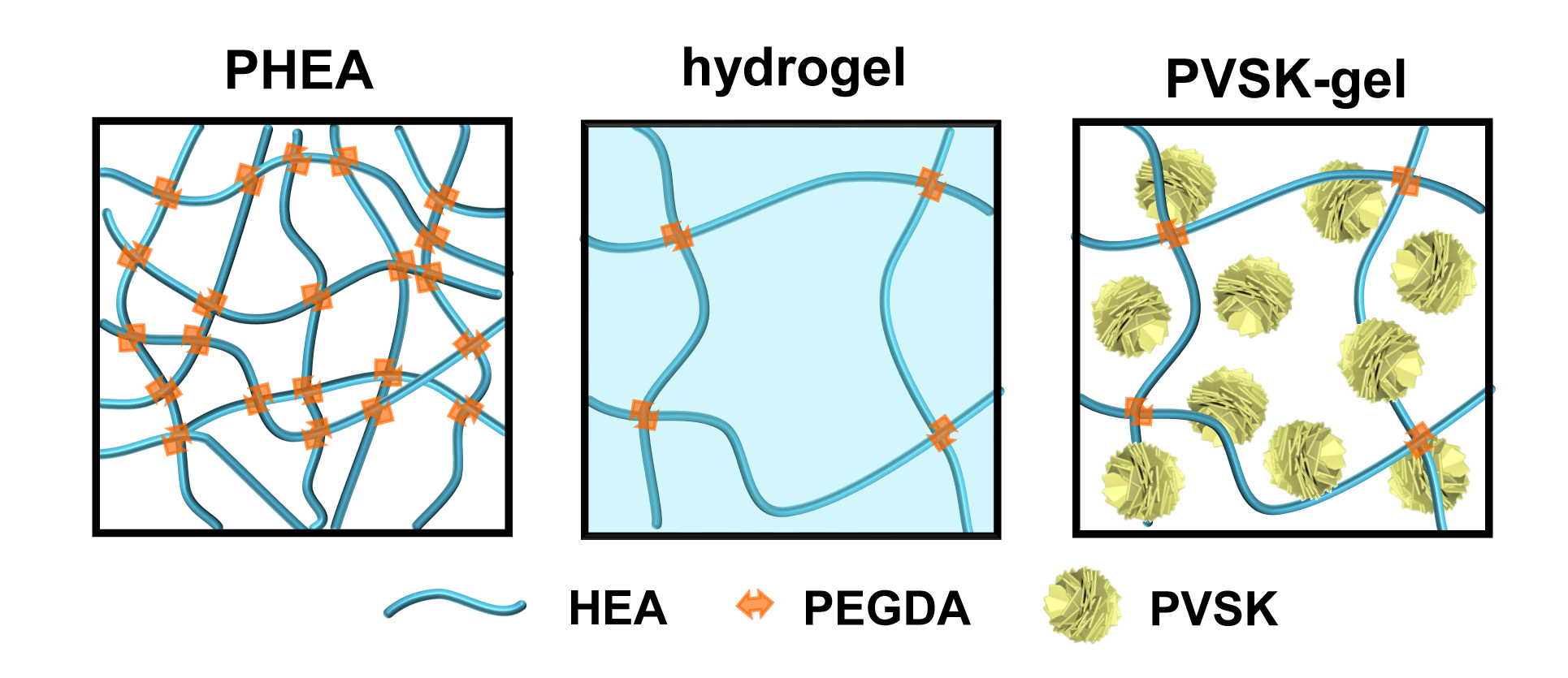


Fig. S17. Schematic illustration of crosslinked networks for PHEA, hydrogel and PVSK-gel. The blue background in the middle picture represents the water surrounding.

Fig. S18. Tan δ and storage modulus curves of the prepared PHEA sample, with a glass transition temperature (Tg) of −8 °C.

Table S1. Preparation parameters of the multicolor perovskite.

| Emission wavelength (nm) | Cs source | Pb source | Annealing temperature (°C) |
| --- | --- | --- | --- |
| 470 | CsBr | PbCl_2_ (0.05 M, 400 mL) | 100 |
| 481 | CsBr | PbCl_2_ (0.05 M, 280 mL) + PbBr_2_ (0.05 M, 120 mL) | 100 |
| 489 | CsBr | PbCl_2_ (0.05 M, 200 mL) + PbBr_2_ (0.05 M, 200 mL) | 100 |
| 499 | CsBr | PbCl_2_ (0.05 M, 120 mL) + PbBr_2_ (0.05 M, 280 mL) | 100 |
| 520 | CsBr | PbBr_2_ (0.05 M, 400mL) | 100 |
| 597 | CsI | PbBr_2_ (0.7 M, 400 mL) | 150 |
| 677 | CsI | PbBr_2_ (0.2 M, 400 mL) | 150 |
| 692 | CsI | PbBr_2_ (0.13 M, 400 mL) | 150 |

Movie S1.

The shape recovery process of the PVSK-gel composites. The movie was 10x speed processed.
